# Supercritical Extraction from Vinification Residues: Fatty Acids, **α**-Tocopherol, and Phenolic Compounds in the Oil Seeds from Different Varieties of Grape

**DOI:** 10.1100/2012/790486

**Published:** 2012-04-19

**Authors:** F. Agostini, R. A. Bertussi, G. Agostini, A. C. Atti dos Santos, M. Rossato, R. Vanderlinde

**Affiliations:** ^1^Laboratório de Óleos Essenciais e Extratos Vegetais, Instituto de Biotecnologia, Universidade de Caxias do Sul, Rua Francisco Getúlio Vargas 1130, Petrópolis, 95070-560 Caxias do Sul, RS, Brazil; ^2^Programa de Pós-Graduação em Botânica, Universidade Federal do Rio Grande do Sul, 91501-970 Porto Alegre, RS, Brazil; ^3^Departamento de Botânica, Instituto de Biociências-UFRGS, Avenue Bento Gonçalves 9500, Bloco IV, Prédio 43433, 91501-970 Porto Alegre, RS, Brazil

## Abstract

Supercritical fluid extraction has been widely employed in the extraction of high purity substances. In this study, we used the technology to obtain oil from seeds from a variety of grapes, from vinification residues generated in the Southern region of the state of Rio Grande do Sul, Brazil. This work encompasses three varieties of *Vitis vinifera* (Moscato Giallo, Merlot, and Cabernet Sauvignon) and two of *Vitis labrusca* (Bordô e Isabel), harvested in 2005 and 2006. We obtained the highest oil content from Bordô (15.40%) in 2005 and from Merlot (14.66%), 2006. The biggest concentration of palmitic, stearic, and linoleic acids was observed in Bordô, 2005, and in Bordô, Merlot, and Moscato Giallo, 2006. Bordô showed the highest concentration of oleic acid and **α**-tocopherol in both seasons too. For the equivalent of procyanidins, we did not notice significant difference among the varieties from the 2005 harvest. In 2006, both varieties Isabel and Cabernet Sauvignon showed a value slightly lower than the other varieties. The concentration of total phenolics was higher in Bordô and Cabernet Sauvignon. The presence of these substances is related to several important pharmacological properties and might be an alternative to conventional processes to obtain these bioactives.

## 1. Introduction

The agribusiness in Brazil produces large amounts of residues. Therefore, the search for alternatives to use organic waste has increased in many research centers. Wine producers have difficulties to dispose of the residual biomass that, though biodegradable, it requires time to be decomposed, thus becoming environment pollutants [1, 2]. However, these residues are becoming attractive for producing high value products [1, 3, 4].

From an ecological point of view, the full utilization of grapes, as byproduct of wine, is an important aspect for reducing waste [5]. This residue is usually burned, used as fertilizer or served as cattle food [3, 6–8]. It is traditionally sold to the oil extraction industries and more recently it has been requested by pharmaceutical and cosmetics industries; for it is a source of antioxidants [9].

The high cost to obtain biologically active natural products is a limiting factor for the economically viable exploration of these resources. As a result, research has been directed to obtain agricultural waste rich in polyphenols [10], fatty acids, and tocopherols [9].

Grape seeds contain an important amount of oil with nutritional potential [8, 11] based on their high level of unsaturated and high content of tocopherols [6, 8]. In addition, the grape seed oil contains tannins in higher concentrations, more than other seed oils [7], such as gallic acid, catechin, epicatechin, and a large amount of procyanidins [12].

The grape seed oil is rich in linoleic acid and *α*-Tocopherol (vitamin E) [13]; in addition, it has high nutritional value, and it can be used as a source of safe-to-eat vegetable oil [4, 5, 7, 14]. The grape seed oil-high in linoleic acid (72–76%), comparing to sunflower seed (60–62%), corn (52%) [15], olive (9–15%) [16], soybean (54%), cotton (53%) [17], canola (22%), and palm (9%) [18], among others.

Vitamin E is an important antioxidant found in vegetable oils [8, 19] that is used by the chemical industries as additives in foods and cosmetics and other uses [20]. Some of the sources of tocopherols on human diet are vegetable oils, fruits, seeds, nuts, cereals, and products derived from them [8, 19]. These compounds are found in small quantities, but they are of great importance due to their medicinal properties. *α*-tocopherol reduces the risk of cardiovascular diseases, diabetes, and cancer and prevents sexual impotence [19–21] and is not synthesized by the human body [20].

Studies have shown that grape seed oil has many pharmacological activities, such as the property of inhibiting the oxidation of low-density lipoprotein [15]. In addition, this oil acts in dissolving thrombi in arteries reducing platelet aggregation and preventing heart attacks, and it acts in the prevention of hypertension and in the normalization of injuries caused by poor circulation due to obesity and diabetes. It may be used for the treatment of obesity, cellulites, and stretch marks, since it helps in the tissue elasticity, reduces swelling and edema, restore collagen and improve peripheral circulation. Additionally, it acts as an excellent antioxidant [9, 13, 15] and shows high nutritional quality for children and the elderly [15]. Today, this oil is increasingly being used in cosmetics and aromatherapy [22].

The extraction of natural products using supercritical fluids has been studied extensively, and it has been used in laboratories both in pilot and industrial scale to isolate substances [23, 24]. When compared to the conventional methods of extraction and separation, supercritical fluids have the characteristics and important advantages that justify the interest in this kind of process. It is potential to solubilize organic compounds of medium and high molecular weight compared to the solubility of the same fluid on steam phase; the low critical temperatures, allowing the extraction of heat-sensitive compounds, causing no alteration in the compound properties. The energy efficiency of the process, operates at low temperatures compared to that of traditional processes, and the solvent/solute easy separation [23, 25, 26]. Moreover, the absence of light and air during extraction can reduce the degradation process common in other techniques [27, 28].

Supercritical CO_2_ is a promising solvent extraction and fractionation of edible oils rich in unsaturated fatty acids, because the extraction can be performed at low temperatures [15]. In addition, the extraction using supercritical fluids offers other positive aspects of traditional techniques such as steam distillation and solvent extraction, due to using a pure solvent that is relatively inexpensive, nonflammable, and nontoxic [15, 24].

Industrial oils seeds are obtained by mechanical pressing and the extraction with organic solvent. During pressing, most of the oil is extracted from the seeds, but a considerable amount remains and a second extraction using a solvent is required for its complete removal. Hexane has been traditionally employed in the extraction, and although it is highly efficient, the oil can suffer thermal degradation and an incomplete solvent residual removal, which are other disadvantages of this process [29].

Just as the cold pressing, the soxhlet extraction uses hexane, resulting in the same problems found in the second stage of cold pressing. These problems are not observed in the supercritical extraction, because the use of organic solvents for oil removal is unnecessary. Another fact favors the use of supercritical extraction: vegetable oil usually must be refined in order to remove not-safe-to-eat compounds. During refining, precious compounds may be lost, thus the supercritical extraction has been suggested as an alternative to obtain enriched oils [25].

Despite the high cost of operation, the supercritical extraction of oil from grape seeds may be more economically viable than the conventional solvent extraction, because the stages of distillation and solvent refining, which increase energy consumption, are not necessary [6, 30].

The aim of this study was to compare the chemical composition of the oil from different varieties of *Vitis* sp., obtained from wine waste from two distinct harvests, through supercritical extraction and chromatographic and spectrophotometric analysis.

## 2. Materials and Methods

### 2.1. Grape Seeds

Three varieties of *Vitis vinifera* (Moscato Giallo, Merlot, and Cabernet Sauvignon) and two varieties of *Vitis labrusca *(Bordô and Isabel) were used in this study. The winemaking wastes were obtained from wineries in the city of Caxias do Sul (Rio Grande do Sul state, South Brazil) and dried at room temperature for seven days. Next, the seeds were manually separated, subjected to moisture, and stored for the tests. They were then ground (20/48 mesh) in a knife mill prior to each experiment.

### 2.2. Supercritical Fluid Extraction on Pilot Scale Equipment (SFE)

A pilot scale equipment (Trendtech) was used for the extraction of the grape seed oil. In these extractions, we used 100 g of ground seeds (20/40 mesh) and the parameters used were 80°C for temperature, CO_2_ flow of 69 g/min and pressure of 250 bar, and the extraction was conducted in 60 minutes.

### 2.3. Analysis of Phenolic Compounds from Seeds Oil of *Vitis* sp. (Folin-Denis)

The determination of phenolic compounds was performed with a liquid-liquid extraction of 1 g of oil and 5 mL of hexane. To this solution we added 6 mL of 60% methanol and the mixture was stirred in a magnetic stirrer for 6 minutes and subsequently transferred to a separating funnel, where the polar phase was drained into a beaker. 5 mL of the polar phase was transferred to a 10 mL volumetric flask, and its volume completed with distilled water. This solution was named polyphenols solution. Quantification of phenolic compounds was performed with the aid of the Folin-Denis using methods described by Roncero et al. [31].

The phenol concentration was estimated by correlation of the samples absorbance with a standard curve made from 1 to 64 *μ*g/mL of gallic acid [12], and the result expressed as mg of gallic acid equivalents/100 g of grape seeds (mg GAE/100 g SU). This curve was conducted in triplicates for three consecutive days.

### 2.4. Analysis of Procyanidins (Hydrolysis with Butanol/HCl) in the Polyphenols Solution of the Seed Oils

According to Porter et al. [32], 6 mL of butanol/HCl (95 : 5) was mixed to 1 mL of polyphenols solution of each variety. To this solution, 0.2 mL of ferric reagent 2% was added. The final solution was shaken and incubated in dry bath for 50 minutes. The absorbance was read at 550 nm.

The procyanidins concentration was estimated by correlating the absorbance of the samples to a standard curve made from 1 to 70 *μ*g/mL of procyanidin B2. The result was expressed as mg of procyanidin B2 equivalents/100 g of grape seed (mg PB2E/100 g SU). This curve was conducted in triplicates for three consecutive days.

### 2.5. Analysis of *α*-Tocopherol by High Performance Liquid Chromatography (HPLC)

Analyses were performed by high performance liquid chromatography (HPLC) HP1100 series, column Lichrospher RP_18_ (5 *μ*m) equipped with UV detector at 294 nm and quaternary pump system, using methanol as mobile phase. The analysis time was 45 minutes and the pattern flow was 0.6 mL/min [21]. The oils were dissolved in chloroform : methanol (2 : 8) at 20 g/L [21] and filtered through nylon membranes of 0.45 pore size. An aliquot of 50 *μ*L of this solution was injected into the chromatograph.

*α*-Tocopherol was identified according to their order of elution and by comparing their retention time with those of pure standard. The quantification was performed by internal standard method, correlating the peak area (mAU*s) to a standard curve made from 0.01 to 9.6 *μ*g/mL of *α*-Tocopherol.

### 2.6. Fatty Acids by Gas Chromatography (GC/MS)

The oils were analyzed by gas chromatography coupled with mass selective detector (GC/MS-Hewlett Packard 6890/MSD5973), equipped with software HP-Chemstation and library spectra Wiley 275. The analyses were performed on HP-INNOWax Polietilenoglicol column (30 m × 250 *μ*m), 0, 25 *μ*m of film thickness (Hewlett Packard, CA, USA), with the following temperature program: 80°C (5 min), 230°C at 3°C/min (30 min), interface 310°C, split ratio (1 : 25), carrier gas He (40 cm/s), acquisition mass range 45–550, injection of 1 *μ*L diluted in hexane (1 : 20).

We performed a quantitative analysis by gas chromatography (GC) Hewlett Packard 6890, equipped with software HP-Chemstation. The analyses were performed on HP-FFAP column (30 m × 250 *μ*m i.d.), 0.25 *μ*m of film thickness (Hewlett Packard, CA, USA), with the following temperature program: 100°C (5 min), 200°C at 5°C/min, 230°C at 3°C/min (30 min); injector temperature 230°C, split ratio (1 : 30), detector temperature FID 240°C, carrier gas H_2_ (59, 3 cm/s) [33]. For the fatty acids quantification was used the standard heptadecanoic acid (C17).

### 2.7. Data Analysis

The data obtained in the oil extraction were subjected to multivariate analysis (AMOVA) and the *t*-test, using SPSS 11.5 program.

## 3. Results and Discussion

### 3.1. Oil Content by SFE (Supercritical Fluid Extraction)

**Figure 1:**
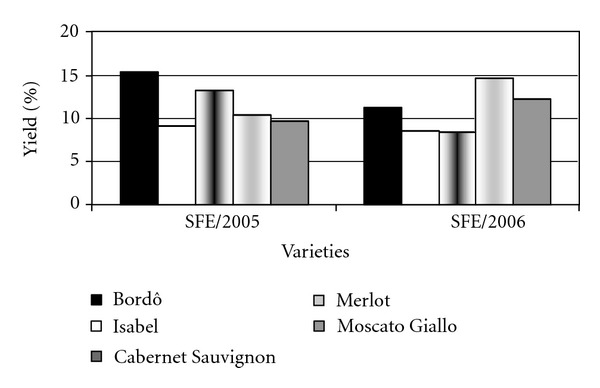
Grape seed oils yield (%w/w) obtained by SFE.

Oil yield (% m/m) is shown in Figure 1, where the values obtained are significantly different. The highest yield was obtained from the variety Bordô (15.40%) in the 2005 harvest and for the variety Merlot (14.66%) in 2006.

Oil yield obtained in this study is in agreement with those reported by Beveridge et al. [13] who evaluated different varieties of *V. vinifera* ranging from 5.85% to 13.60%, using 370 bar, 65°C and CO_2_ flow rate of 60 g/min and 6 h for extraction. The pressure and extraction time used by those authors were higher than those employed in the present study. However, the temperature was lower. Bravi et al. [34] obtained maximum oil yield of 14.40% from mixed seeds of different varieties of *V. vinifera*, using 250 bar and 40°C for 7 h of extraction, which is in agreement with the levels obtained in this study. The temperature used was higher than that used by these authors, but the extraction time was lower and the pressure was the same.

The oil yield obtained in this study was higher than that described by Gómez et al. [6] who extracted oil from the seeds of the Airen type, using 350 bar and 40°C for 3 h, obtaining a yield of 6.90%. Comparing the extraction conditions, we used higher temperature and lower pressure and extraction time.

Cao and Ito [15] also obtained a yield lower than what we obtained in this study (6.20%) from grape seeds of a variety not described, using 400 bar and 40°C during 3 h. These authors used higher pressure and longer extraction time at lower temperature, different than the method we employed in this work.

The observed differences may be due to several factors, such as pressure, temperature, extraction time, and the variety of grape used in different studies. The solubility of seeds oil normally increases in raising pressure due to enhanced density of the supercritical fluid [4, 35], also the raise in temperature causes an increase in vapor pressure of the solute, reducing the supercritical fluid density, thus magnifying the oil yield extracted [35].

According to Cao and Ito [15], the pressure was the most important factor in the extraction of grape seed oil because higher pressures increase significantly the oil yield and the amount of unsaturated fatty acids extracted. However, the temperature did not influence the oil content. The high yields we obtained in this study are due to the different varieties we studied. The yields were higher than those obtained by Gómez et al. [6] and Cao and Ito [15], who used higher pressures.

### 3.2. Fatty Acids, *α*-Tocopherol, Total Phenolics, and Procyanidins

**Figure 2:**
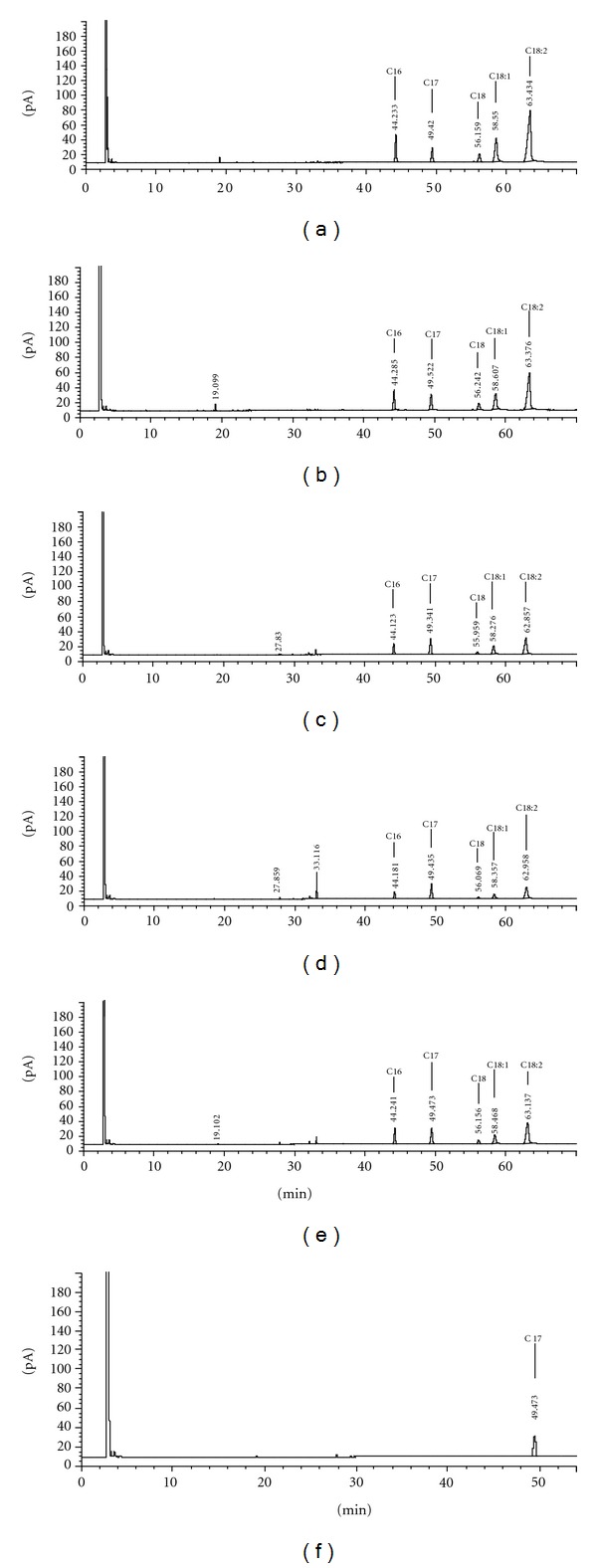
Chromatograms of fatty acids extracted from grape seeds oil from the varieties Bordô (a), Isabel (b), Cabernet Sauvignon (c), Merlot (d), and Moscato Giallo (e). Heptadecanoic acid standard-C17 (f).

The fatty acid composition is shown in Table 1 and Figure 2, where four fatty acids are in evidence: palmitic (C16), stearic (C18), oleic (C18 : 1), and linoleic (C18 : 2). The results are in agreement with different authors [6, 13, 15, 29, 34, 35] who also demonstrated the presence of these fatty acids in grape seed oil extracted by SFE.

**Figure 3:**
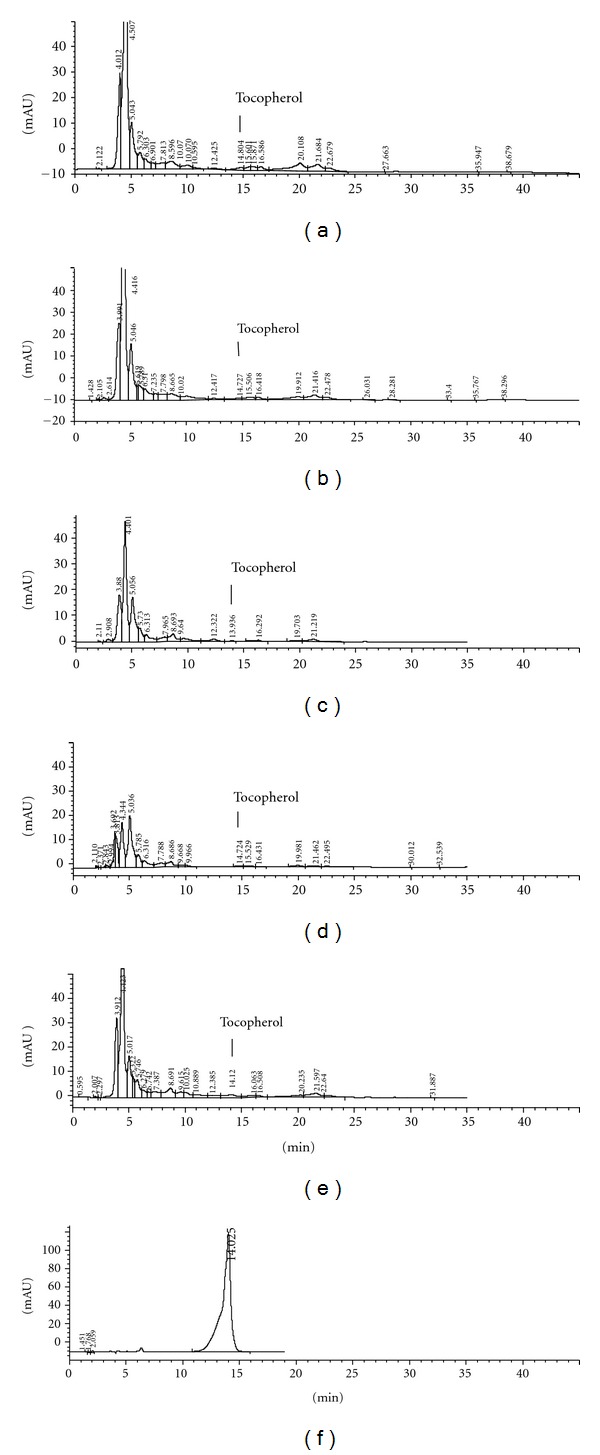
Chromatograms of *α*-Tocopherol extracted from grape seeds oil from the varieties Bordô (a), Isabel (b), Cabernet Sauvignon (c), Merlot (d), and Moscato Giallo (e). *α*-Tocopherol standard (f).

The oils were also analyzed for the presence of *α*-Tocopherol (Figure 3) by high performance liquid chromatography (HPLC), using the standard *α*-Tocopherol, which showed a standard curve with linear regression coefficient (R²) of 0.9997.

Regarding fatty acids (Table 1), in general, there was an increased concentration in the varieties Merlot and Moscato Giallo between harvests and between Isabel and Bordô; there was a reduction in the stearic acid, and palmitic acid was reduced only in Bordô. 

The largest concentration of palmitic, stearic, and linoleic acids was observed in the Bordô, 2005 variety, and for Bordô, Merlot and Moscato Giallo, 2006. Bordô also showed the highest concentration of oleic acid in both seasons.

The variations found in the fatty acids concentrations may be due to indirect effects of solar radiation, air temperature, rainfall, and humidity as suggested by Mandelli [36]. These elements play a major role in the development, production, maturation, and quality of the grape in the region. These factors may have influenced the metabolism of vines and provided the variations observed in the concentrations of fatty acids in relation to varieties evaluated in this study.

The major compound found in the oil, obtained by SFE, from the varieties here studied was the linoleic acid and its in agreement with other authors [6, 13, 15, 29, 34, 35]. According to Sahena et al. [26], the fatty acids solubility in supercritical CO_2_ depends on the size of the hydrocarbons chain and the presence of functional groups, as well as the effect of extractions parameters such as pressures and temperature.

The variety Bordô, still little studied in Southern Brazil, showed the highest concentrations of fatty acids in comparison to other varieties and demonstrated high potential for oil extraction. Estimating the oil production versus the amount of grapes processed in Rio Grande do Sul in 2005, using the average of oil yield obtained by SFE in this study and the percentage of grape seeds present in grape marc, we obtained in 2005: 494,299.7 kg of Isabel oil and 257,018.3 kg of Bordô oil, 41,848.5 kg of Cabernet Sauvignon oil, 39,966.3 kg Merlot oil, and 2,319.4 kg of Moscato Giallo oil, highlighting the potential of Isabel and Bordô for oil production.

In respect to *α*-Tocopherol (Table 1), we observed an increase in the varieties Merlot and Moscato Giallo, between harvests, and a reduction in the variety Bordô, which showed the highest concentration of this compound in both seasons.

These data suggest that several facts are influencing the amount of *α*-Tocopherol, such as the grape variety used for the oil extraction or the variation in the conditions of supercritical extraction, in agreement with Bravi et al. [34], who argues that the amount of *α*-Tocopherol extracted by SFE increased with the temperature.

The procyanidins equivalents (Table 1) showed an increase only in Bordô, since the other varieties showed no significant differences between harvests. In the 2005 harvest there was no significant difference among the varieties studied; in the 2006 harvest Isabel and Cabernet Sauvignon showed a value slightly lower when compared to other varieties studied.

The concentration of total phenolic compounds present in the oils of the varieties showed no significant changes (Table 1), but there was a higher concentration of equivalents of gallic acid in the varieties Bordô and Cabernet Sauvignon, 2005, and in Bordô, Cabernet Sauvignon and Merlot in 2006.

Grapes are considered a major source of phenolic compounds comparing to other fruits and vegetables, but the great genetic diversity among varieties results in grapes with different characteristics, flavor, and color, which is associated with content and profile of polyphenols [37]. The concentration of phenolic compounds depends on the vine variety and it is influenced by environment factors [38, 39]. Probably these are the major factors causing the differences found in this study, regarding phenolic compounds. As to the data obtained in the present study, the variety Bordô is in evidence for having high concentrations of fatty acids, procyanidins, total phenolics, and *α*-Tocopherol.

## Figures and Tables

**Table 1:** Fatty acids, *α*-Tocopherol, total phenolic, and procyanidins concentration (mg/100 g of grape seeds), extracted from the different varieties of grapes from 2005 and 2006 harvests.

			Compounds concentration/100 g of grape speed							
Chemical compound	Bordô		Isabel		Cabernet sauvignon		Merlot		Moscato Giallo	
	2005	2006	2005	2006	2005	2006	2005	2006	2005	2006
C16^1^	496.93Aa	316.87Ba	185.43Ab	174.28Ab	149.58Abc	111.03Ab	77.95Bc	281.14Aa	156.61Bbc	273.32Aa
C18^2^	194.68Aa	147.62Ba	85.07Ab	29.82Bb	43.90Abc	23.43Ab	23.71Bc	134.89Aa	55.11Bbc	149.30Aa
C18 : 1^3^	774.37Aa	713.37Aa	280.67Ab	202.40Ac	187.03Abc	62.83Ad	79.20Bc	430.62Ab	143.06Bc	466.55Ab
C18 : 2^4^	2315.24Aa	2162.82Aa	800.36Ab	243.44Ab	425.65Ab	113.49Ad	252.73Bb	1951.18Aa	427.25Bb	2059.05Aa
Proanthocyanidins^5^	0.31Ba	0.54Aa	0.19Aa	0.25Aab	0.31Aa	0.25Aab	0.21Aa	0.37Aa	0.21Aa	0.39Aa
Phenolics^6^	2.37Aa	2.36Aa	1.70Ab	1.25Ab	2.13Aa	2.06Aa	1.57Ab	01.74Aa	1.33Ab	1.23Ab
*α*-Tocopherol^7^	1.32Aa	0.65Ba	0.39Ac	0.24Ab	0.26Ac	0.14Abc	0.17Bcd	0.37Ab	1.12Ab	0.18Bbc

Means followed by the same letter do not differ significantly from each other by AMOVA 0.05%.

Capital letters correspond to the lines in columns between different varieties and the tiny letters correspond to the lines in the columns of the same year (refer to compounds). ^1^Palmitic acid (mg/100 g grape seed); ^2^stearic acid (mg/100 g grape seed); ^3^oleic acid (mg/100 g grape seed); ^4^linoleic acid (mg/100 g grape seed), ^5^mg procyanidin B2 equivalent/100 g of grape seed; ^6^mg gallic acid equivalent/100 g of grape seed, ^7^mg/100 g of grape seed.
